# Best Evidence Rehabilitation for Chronic Pain Part 4: Neck Pain

**DOI:** 10.3390/jcm8081219

**Published:** 2019-08-15

**Authors:** Michele Sterling, Rutger M. J. de Zoete, Iris Coppieters, Scott F. Farrell

**Affiliations:** 1 RECOVER Injury Research Centre, The University of Queensland, 4006 Brisbane, Australia; 2 NHMRC Centre of Research Excellence in Road Traffic Injury Recovery, The University of Queensland, 4006 Brisbane, Australia; 3 Menzies Health Institute Queensland, Griffith University, 4222 Gold Coast, Australia; 4 Pain in Motion International Research Group, Department of Physiotherapy, Human Physiology and Anatomy, Faculty of Physical Education & Physiotherapy, Vrije Universiteit Brussel, 1090 Brussels, Belgium; 5 Department of Rehabilitation Sciences and Physiotherapy, Faculty of Medicine and Health Sciences, Ghent University, 9000 Ghent, Belgium

**Keywords:** neck pain, rehabilitation, exercise, psychology review

## Abstract

Neck pain, whether from a traumatic event such as a motor vehicle crash or of a non-traumatic nature, is a leading cause of worldwide disability. This narrative review evaluated the evidence from systematic reviews, recent randomised controlled trials, clinical practice guidelines, and other relevant studies for the effects of rehabilitation approaches for chronic neck pain. Rehabilitation was defined as the aim to restore a person to health or normal life through training and therapy and as such, passive interventions applied in isolation were not considered. The results of this review found that the strongest treatment effects to date are those associated with exercise. Strengthening exercises of the neck and upper quadrant have a moderate effect on neck pain in the short-term. The evidence was of moderate quality at best, indicating that future research will likely change these conclusions. Lower quality evidence and smaller effects were found for other exercise approaches. Other treatments, including education/advice and psychological treatment, showed only very small to small effects, based on low to moderate quality evidence. The review also provided suggestions for promising future directions for clinical practice and research.

## 1. Introduction

The health and economic burdens due to neck and back pain are substantial, being the leading cause of years-lived-with-disability worldwide [1]. The societal burden of these conditions is driven by the fact that many cases do not recover from acute episodes but go on to develop persistent or recurrent pain [2,3]. Neck pain may arise as a consequence of traumatic injury, usually a motor vehicle crash (whiplash associated disorder—WAD) or be of a non-traumatic onset such as occurs in office workers (termed ‘non-traumatic neck pain’ in this review). Some argue that there is little difference between the two neck pain conditions and have developed classification systems that do not differentiate between them [4]. However, direct comparisons between WAD and non-traumatic neck pain have found the former group report higher levels of pain and disability [5], greater psychological distress [5], more marked hyperalgesia and hypoesthesia indicative of nociplastic pain [6,7], and have worse outcomes at follow-up [8]. These findings suggest that different mechanisms may underlie WAD and non-traumatic neck pain and subsequently different classification systems and treatments may be necessary depending on the patient presentation.

The aim of this review is a state-of-the-art overview of the best evidence rehabilitation for patients with neck pain. The best evidence rehabilitation is reviewed in a way that clinicians can integrate the evidence into their daily clinical routine. In addition, the state-of-the-art overview also serves clinical researchers to build upon the best evidence for designing future trials, implementation studies, and to develop new innovative studies.

## 2. State-of-the-Art

For this best evidence review, rehabilitation was defined as aiming to restore a person with neck pain to health or normal life through training and therapy (Oxford dictionary definition). It will include a review of active non-interventional treatments such as exercise, psychology, and multimodal approaches. Interventional procedures (e.g., radio-frequency neurotomy), pharmaceutical treatment, and passive treatments—such as acupuncture or manual therapy alone and not used in conjunction with more active treatments—will not be included. For this review, a non-systematic search of scientific studies was performed in MEDLINE (PubMed), Scopus, CINAHL, and Pedro using the following search terms: chronic neck pain, neck pain, whiplash associated disorders, rehabilitation, exercise. To minimize selection bias and to ensure high quality evidence was selected, systematic reviews and meta-analyses were preferred and sought where possible. Recent high quality randomised clinical trials (RCTs) not already included in systematic reviews were included as well as information from large population-based cohorts and international clinical guidelines. High quality trials were defined as those receiving a Pedro score of 6/10 or greater and Pedro scores are provided for each trial included in this review. Both WAD and non-traumatic neck pain were included with the aim of separating the results for both neck pain groups where possible. This review did not include cervical radiculopathy.

A summary of the results are provided in Table 1.

## 3. Reassurance, Advice, and Education

It is acknowledged that the provision of reassurance, advice, and education are subtly different paradigms, but as they are often difficult to study in isolation, they have been included together for the purposes of this review.

Providing advice and reassurance to the patient with neck pain is the first step in the rehabilitation process and is often the first-line treatment recommended by clinical practice guidelines [24,25]. Currently, there is no clear guidance on the recommended content of reassurance, beyond the message of a favourable prognosis and full recovery [24]. In a recent qualitative study, the views of both patients with WAD and non-traumatic neck pain were sought about the issues that concerned them most. We found that both groups of patients wanted similar kinds of information that were consistent with themes of worry about possible undetected structural damage; distress about difficulty undertaking usual activities; concerns about the future and hardships such as treatment costs and insurance claims [26]. These findings may provide some direction to the nature of reassurance required by patients with neck pain.

Various information and educational approaches including information booklets, websites, and videos have been investigated for their effectiveness in improving outcomes following whiplash injury [9]. This Cochrane review identified three overall themes for patient education: advice on increasing activity, advice focusing on pain and stress coping skills, and workplace ergonomics and advice on self-care strategies. The review found that an educational video of advice in the hospital Emergency Department that focussed on resuming activity was more beneficial in decreasing acute WAD symptoms than no treatment at 24 weeks follow-up (RR 0.79, 95% CI: 0.59 to 1.06) but not at 6 and 52 weeks. The number of patients who must receive this educational video intervention for one to benefit was 23. The results were based on one RCT only. No other educational intervention was found to be effective, including a WAD information pamphlet provided in ED [9]. Later systematic reviews have found that structured patient education alone did not yield large benefits in clinical effectiveness compared with other conservative interventions for patients with WAD or non-traumatic neck pain [10,11].

Recent RCTs not included in the above-mentioned reviews have found similar results. An educational treatment of pain management focusing on understanding/acceptance of pain, goal setting, and participation in social- and work-related contexts was less effective in improving a physical quality of life measure when compared to a multimodal treatment (the same education in addition to exercise) for a mixed traumatic/non-traumatic neck pain sample [27] (Pedro 6/10). A preliminary RCT in a small sample of women with mixed chronic neck pain also found that pain education was less effective when delivered alone than when delivered in conjunction with exercise [28] (Pedro 7/10). 

Education directed toward increasing a patient’s knowledge of pain neuroscience has gained traction in recent years, mostly in the field of low back pain. Systematic reviews have found that this approach has a small to moderate effect on pain and disability in the short-term immediately following the intervention for patients with low back pain [29]. There is also moderate level evidence that the use of pain neuroscience education alongside other physiotherapy interventions probably improves disability and pain in the short term in chronic low back pain [30]. Pain neuroscience has been less investigated in neck pain, with one preliminary RCT showing potential benefit when combined with exercise for adolescents with non-traumatic neck pain [31] (Pedro 7/10). 

Clinical message: Tested educational/advice treatments alone show only small effects for patients with neck pain (WAD and non-traumatic neck pain). Better effects may be seen when educational approaches are used in conjunction with exercise. The optimal content to be included in educational approaches is not known.

## 4. Exercise

Various types of exercise have been evaluated for their effectiveness in neck pain, including general exercise and activity, neck specific strengthening or control exercises, and sensorimotor exercises. Systematic reviews generally include all exercise types together. A recent comprehensive Cochrane systematic review found no high quality evidence, indicating that there is still uncertainty about the effectiveness of exercise for neck pain [12]. In an attempt to gain clarity around the effectiveness of exercise alone, this review included only trials with single interventions that compared exercise with a control or comparative group. Additionally, the authors used an exercise classification system based on a clinical rationale for selecting studies with similar interventions to assist with interpretation and inclusion within the meta-analyses [12].

Moderate quality evidence supported the use of upper quarter (neck, scapula, and upper limb) strength training to improve pain immediately post treatment with a moderate to large effect (pooled SMD (pain) −0.71 (95% CI: −1.33 to −0.10)) at short-term follow-up. There was also moderate quality evidence to support: (i) upper-quarter endurance training for a small beneficial effect on pain immediately post treatment and short-term follow-up; (ii) neck and shoulder girdle muscle control (stabilisation) exercises to improve pain and function at intermediate term follow-up (MD pain (100 point scale) −14.90 (95% CI: −22.40 to −7.39)); and (iii) Qigong (mindfulness and slow movement exercise) to minimally improve function but not global patient perceived effect in the short term. Low quality evidence suggested that breathing exercises; general fitness training; stretching alone; and vestibular rehabilitation type exercises may not change pain or function at immediate post treatment to short-term follow-up. Very low quality evidence suggested that neuromuscular eye–neck co-ordination/proprioceptive exercises may improve pain and function at short-term follow-up, supporting findings of a previous systematic review [16].

From this review, it should be noted that the best available evidence is at a moderate quality level, meaning that further research is likely to have an important impact on the effect estimate. The review also did not differentiate between WAD and non-traumatic neck pain, but there may well be different responses to exercise (and other treatments) between the two neck pain groups as a consequence of their different clinical presentation and features outlined earlier in this review. A later review found similar results, concluding that supervised qigong, yoga, and combined programs including strengthening, range of motion, and flexibility are effective for the management of persistent neck pain, with no one program superior to another [32]. These authors also noted that effect sizes are small indicating a small clinical benefit of exercise for chronic neck pain [32].

Other systematic reviews have investigated the evidence for one specific exercise type. Exercises to improve control of the cranio-cervical flexion movement were found to have small to moderate effect on pain in the short to intermediate term (SMD pain −0.59 (95% CI: −0.97 to −0.20)) and a small effect on disability (SMD disability −0.44 (95% CI: −0.81 to −0.08)) when compared to other treatments (other exercise, manual therapy) in people with non-traumatic neck pain [14]. However, the meta-analysis revealed high heterogeneity indicating that results should be interpreted with caution. Another systematic review without meta-analysis found that motor control exercises may not be any more effective than a standard strengthening exercise program [33]. Yoga was found to have moderate positive effects on pain (SMD −1.13 (95% CI:−1.60 to −0.66)) and disability (SMD = −0.92 (95% CI: −1.38 to −0.47)) over other treatments (mostly other exercise) for people with chronic non-traumatic neck pain [17] but the results had high heterogeneity, indicating caution is required with interpretation.

Two systematic reviews have evaluated the effect of general exercise and activity for neck pain with both reviews acknowledging the limited number of trials available for inclusion. One review found that there were no clinically meaningful differences between comprehensive exercise programs, which included general exercise, and minimal intervention controls in the medium and long term [18]. A second review including studies of both non-traumatic neck pain and WAD found small effects on pain that were probably not clinically significant [34].

Clinicians may want to understand if one form of exercise is more effective than another. While all systematic reviews note small effects for most exercise types, it has been commonly assumed that there is no difference between them. However, few direct head-to-head comparisons of different exercise types have been undertaken and therefore firm conclusions cannot be drawn. Some data suggest differential effects of exercise type on pain sensitivity. For example, isometric exercise may exert greater hypoalgesic effects than aerobic exercise, at least in the short term [35], so it is possible that further research may show that exercise type has different influences on pain.

Clinical message: Exercise seems to have beneficial effects on neck pain (WAD and non-traumatic neck pain), although high quality evidence is lacking. There is moderate evidence indicating that strengthening exercises of the upper quarter may have a moderate effect on pain, but further research may change this result. At present, there is no data available to show that one form of exercise is more effective than another. There is also no data to indicate the optimal dose or intensity. Until further evidence becomes available, clinicians may want to take patient preference and their clinical expertise with exercise prescription into account when providing an exercise intervention. They should also consider the potential overall health benefits of exercise (particularly aerobic and strengthening exercises).

## 5. Work Place Neck Pain

Office workers have the highest annual prevalence of neck pain (up to 63% depending on neck pain definition) of all occupations [36]. Neck pain is a recurrent condition with 60–80% of workers reporting a recurrence one year after the initial episode [37]. Ergonomic interventions such as adjustments to the physical work space and equipment are commonly employed, with the aim of reducing physical strain to the musculoskeletal system, thus reducing risk of injury. A recent Cochrane review found inconsistent evidence that the use of an arm support or an alternative mouse may reduce the incidence of neck and should disorders (risk ratio (RR) 0.52 (95% CI: 0.27 to 0.99)). For other physical ergonomic interventions, they found no evidence of an effect and for organisational interventions, such as increased work breaks, very low-quality evidence was found for an effect on the incidence of upper limb pain [38]. Another recent systematic review found low quality evidence that ergonomic programs do not reduce the risk of a new episode of neck pain (OR 1.00 (95% CI: 0.74 to 1.35)) but moderate quality evidence that an exercise program substantially reduces the risk of a new episode of neck pain (OR 0.32 (95% CI: 0.12 to 0.86)) [39]. The latter finding was based on two RCTs, one included aerobic, strengthening, and stretching exercises and the other strengthening and stretching exercises [39]. 

For workers with neck and/or upper limb pain, another Cochrane review found very low quality evidence that exercise interventions were no more effective than no treatment on pain disability and sick leave [40]. This review also found that ergonomic interventions did not decrease pain in the short term but did decrease pain in the long term (low quality evidence) [40]. An earlier review found moderate quality evidence that a multiple-component intervention (including mental health education, physical health education, relaxation and breaks, activity modifications, and physical environmental modifications) reduced sickness absence in the intermediate-term (OR 0.56 (95% CI: 0.33 to 0.95)) which was not sustained over time but no intervention had an effect on pain [41]. However, a more recent review and meta-analysis concluded that workplace-based strengthening exercises were effective in reducing neck pain in office workers with a larger effect if the exercises were targeted to the neck/shoulder with moderate quality evidence (SMD pain = 0.59 (95% CI 0.29 to 0.89)) [13]. There was a dose–response relationship with greater participation in the exercise being associated with a larger effect [13]. These conclusions were supported by another review that concluded that there is level II evidence recommending that clinicians include strengthening exercise to improve neck pain and quality of life [42]. Recommendations arising from all reviews were that further large high quality clinical trials are needed.

Since the publication of these reviews, several RCTs have investigated various interventions for workplace related neck pain. Neck and shoulder stretching exercise and ergonomic advice improved neck pain and disability to a greater extent than ergonomic advice alone, with small effects (−1.4; 95% CI: −2.2 to −0.7 for visual analogue scale; −4.8; 95% CI: −9.3 to −0.4 for Northwick Park Neck Pain Questionnaire) in workers with at least moderate neck pain (Pedro 8/10) [15]. However, Caputo and colleagues found no difference between a twice-weekly 7-week group-based neck and shoulder resistance exercise programme compared to group-based stretching and postural exercise of the same duration [43] (Pedro 7/10).

Clinical message: Work-place strengthening programs may be effective for office workers with non-traumatic neck pain and for preventing neck pain in asymptomatic workers. Evidence for the effectiveness of ergonomic interventions is inconsistent.

## 6. Psychological Treatments

Similar to all chronic pain conditions, neck pain is associated with psychological factors such as cognitive distress, anxiety, depressed mood, fear of pain and/or movement [44,45] and in the case of WAD, posttraumatic stress symptoms [45]. Psychological factors likely play a role in the transition from acute to chronic pain [46] and contribute to the extent and severity of pain and disability reported [46]. Treatments directed at psychological factors can decrease pain and disability [47].

Cognitive behavioural therapy (CBT) is one of the most common psychological treatments used in the treatment of chronic pain conditions. CBT works by means of modifying maladaptive and dysfunctional thoughts (e.g., catastrophising, kinesiophobia) and improving mood (e.g., anxiety and depression), leading to gradual changes in cognition and illness behaviour. With respect to neck pain, a recent Cochrane review found the quality of the evidence to be very low to moderate with only six high quality RCTs being available for inclusion [19]. CBT was found to be more effective for short-term pain (SMD pain −0.58 (95% CI: −1.01 to −0.16)) and disability (SMD disability −0.61 (95% CI: −1.21 to −0.01)) reduction only when compared to no treatment. There was moderate quality evidence that CBT was better than other interventions for improving kinesiophobia at intermediate-term follow-up (SMD −0.39 (95% CI: −0.69 to −0.08)). For subacute neck pain, CBT was significantly better than other types of treatment at reducing pain in the short-term (SMD pain −0.24 (95% CI: −0.48 to 0.00)), but there were no effects on disability and kinesiophobia. Looking at psychological treatments more broadly, Shearer and colleagues found no evidence for or against the use of psychological interventions (including relaxation training and CBT) in patients with recent onset neck pain or WAD [48]. For chronic neck pain, they found evidence that a progressive goal attainment program may be helpful.

The results of both systematic reviews illustrate the dearth of clinical trials investigating psychological treatments for neck pain with only 10 RCTs eligible for inclusion. This is in comparison to say low back pain, where systematic reviews have included up to 30 RCTs [49,50].

Clinical message: There is little evidence available to determine if psychological treatments alone are effective for neck pain (WAD and non-traumatic neck pain).

## 7. Combined Treatments—Physical and Psychological Treatments

Many clinical trials have investigated interventions of mixed treatments and or disciplines. The most recognised of these approaches is multidisciplinary rehabilitation. No systematic reviews for multidisciplinary rehabilitation of chronic neck pain were located. A review of this approach for chronic low back pain found modest positive effects on pain and disability compared to usual care or physical rehabilitation [51].

A more common approach studied in patients with neck pain is to add a psychological treatment to a physiotherapy program with both components delivered by physiotherapists. A recent systematic review and meta-analysis of this combined approach found that, for neck pain and WAD, there was no effect on pain or disability but statistically and probably clinically relevant effects on fear of movement beliefs (SMD −0.5 (95% CI: −0.95 to −0.05)) and pain catastrophizing (SMD −0.31 (95% CI: −0.54 to −0.08)) [20]. However, the meta-analysis revealed high heterogeneity, potentially as the psychological interventions and usual physiotherapy/usual care were not uniform across the included RCTs. The definition of psychological intervention in this review was kept broad. Studies with larger effects were more closely examined, revealing that these RCTs tended to use individually tailored interventions. In addition, these interventions addressed patients’ maladaptive cognition through the use of various cognitive techniques (e.g., identifying and challenging negative thoughts) while aiming to modify patients’ maladaptive behaviours and increasing level of activities using a range of behavioural strategies (e.g., breathing and relaxation techniques, goal setting, and graded activities). Furthermore, some of these studies also encouraged patients to participate in the decision making regarding goal settings and treatment. One of these studies included patients with WAD [52], with the other being of patients with low back pain [53].

Since this review, one additional RCT investigating effects of combined psychological/physiotherapy interventions has been published, all with slightly different approaches. A recent RCT (StressModex) showed that a physiotherapist delivered exercise program and a psychological treatment targeting initial stress related symptoms in patients with acute WAD and at high risk of poor recovery, was more effective than exercise alone on pain related disability (primary outcome) (Neck Disability Index at 6 weeks: −10.0 (−15.5 to −4.8); at 6 months: −7.8 (−13.8 to −1.8) and at 12 months: −10.1 (−16.3 to −4.0)) and stress, depression and self-efficacy (secondary outcomes) [21]. The effect size on the primary outcome was moderate to large. The psychological component of the combined intervention was consistent with that outlined in the above systematic review [20]—it was a targeted intervention using cognitive strategies and behavioural strategies. Whilst there has been no direct head to head comparison, the results of StressModex are superior to what has been found for both ‘psychologically informed’ physiotherapy (MINT trial) [54] and early multidisciplinary care [55] for acute WAD. In both these trials, there was no stratification of patients based on risk to recovery and treating all patients as a homogenous group may dilute any treatment effect. The former trial used less targeted strategies for dealing with psychological factors where the physiotherapists questioned patients to identify treatment targets, such as beliefs about pain and coping strategies. It is possible that the approach used in the MINT trial was too broad and although attempted to address psychosocial factors, lacked the specificity to be effective. StressModex specifically targeted one psychological risk factor and trained physiotherapists in its management as opposed to a more broad approach and this may be a reason for the stronger effects seen. In the latter multidisciplinary trial, patients were less compliant with psychology treatment compared to physiotherapy. Patients in the acute stage of a physical injury may not see the relevance of seeing a psychologist for what they perceive is a physical injury. This may be further justification for an enhanced role of physiotherapists in the management of acute WAD (and other musculoskeletal injuries), with that role including the management of psychological aspects of the condition in addition to physical ones.

With respect to non-traumatic neck pain, few trials have tested a combined physiotherapy/psychological intervention. One RCT of fair quality (Pedro 5/10) found no added benefit of a cognitive behavioural intervention to exercise on disability but clinically meaningful reductions in pain [56].

Taken together, the results of these studies suggest that psychological treatments delivered by physiotherapists may need to be individually targeted (personalized rehabilitation) as opposed to a broad psychologically-informed approach. Further research is required to determine the important and effective components of a physiotherapist-delivered psychological intervention.

Clinical message: Combined psychological and physiotherapy interventions delivered by physiotherapists may be more effective than physiotherapy alone for WAD but these results need further replication. The psychological component may be more effective if it specifically targets individual psychological factors. 

## 8. Combined Treatments—Exercise and Passive Treatments

Manual therapy is a common treatment provided to patients with neck pain. It was not an aim of this review to synthesise evidence for passive treatments used in isolation. However, as manual therapy is often combined with exercise in clinical practice, it is worthwhile to consider if its addition has any greater effects than exercise alone. A systematic review of RCTs including both WAD and non-traumatic neck pain concluded that combined manual therapy and exercise was no more effective than exercise alone in reducing neck pain intensity, neck disability, or improving quality of life [22]. In contrast, Hildago et al. [57] found moderate evidence supporting combined manual therapy and exercise for acute to sub-acute neck pain and moderate to strong evidence for chronic neck pain. In their systematic review, Sutton and colleagues [58] found that multimodal care—including education, exercise, and manual therapy—can benefit patients with WAD and non-traumatic neck pain. They also concluded that there is no additional benefit to providing frequent sessions of multimodal care to patients with neck pain over an extended time period [58]. 

Clinical message: Adding manual therapy to exercise may be more beneficial than exercise alone, but the evidence is conflicting.

## 9. Lifestyle Interventions

In recent times, attention has been paid to the potential role of lifestyle factors in the development of chronic musculoskeletal pain. Epidemiological studies have found that higher levels of physical activity are associated with less neck and shoulder pain [59] and physical inactivity and high Body Mass Index are associated with an increased risk of chronic pain in the low back and neck/shoulders in the general adult population [60]. Sleep problems have also been shown to be associated with an increased risk of chronic pain in the low back and neck/shoulders [61]. These data suggest that interventions directed at improving lifestyle factors may be effective for musculoskeletal pain, including neck pain, but few trials have been conducted in this area. Williams and colleagues found that a 6-month telephone-based healthy lifestyle coaching service intervention provided no benefit (primary outcome was pain) over usual care for obese patients with low back pain [62] (Pedro 8/10). The intervention was not successful in changing the targeted lifestyle factors such as weight, physical activity, diet, and sleep and this may explain the lack of effect on pain [62]. No recent trials of lifestyle interventions for neck pain were found but this could be an area of future research.

Clinical message: The effect of lifestyle interventions on chronic neck pain is unknown as no studies have yet been conducted. 

## 10. Patient Stratification and Sub-Grouping

The mostly small effects seen in clinical trials for musculoskeletal pain conditions have led to suggestions that the small effects are due to the heterogeneity of the conditions and their subsequent differential response. In response, there has been much debate about the merits or otherwise of sub-grouping patients which may in turn lead to the identification of the most effective treatment for each sub-group [63]. Some RCTs, using patient sub-grouping, have been conducted in low back pain with limited effects seen [64]. With respect to neck pain, few studies looking at the benefits and effects of patient sub-grouping have been conducted and all have been in WAD. In a preliminary RCT, a neck and shoulder girdle specific exercise program was found to be more effective in patients with chronic WAD and signs of central sensitisation (widespread mechanical and cold hyperalgesia) when compared to those without these features [65] (Pedro 7/10). However, in this trial, the sub-group analyses were not pre-defined and prior sample size calculations did not consider sub-group analyses, indicating that the results should be interpreted with a high degree of caution. In a later trial with a priori sample size calculations that included investigation of the moderating effects of several variables on the outcomes, the results were not replicated [66]. No evidence was found that measures of central sensitisation or psychological variables of posttraumatic stress symptoms moderated the effect of a 12-week exercise intervention in patients with chronic WAD [66]. Similarly, in patients with acute WAD (<4 weeks post injury) providing different treatments based on the individual patient’s sensory and psychological presentation provided no better effect than usual care [55]. In this RCT, if patients presented with widespread hyperalgesia and significant psychological distress, they received physiotherapy exercise, medication, and psychological treatment, whereas those without these features received physiotherapy exercise alone. This pragmatic approach using patient sub-grouping was compared to usual care [55]. In summary, sub-grouping patients with WAD based on sensory and psychological measures has not yet been successful.

Another approach has been to stratify patients, usually those with an acute condition, based on their risk of recovery or non-recovery. Again, most research in this area has been in WAD. A clinical prediction rule to identify both chronic moderate/severe disability and full recovery at 12 months post-injury was recently developed [67,68]. The results indicated that an initial Neck Disability Index score of ≥40%, age ≥35 years, and a score of ≥6 on a hyperarousal symptom scale (symptoms of trouble falling asleep, irritability, difficulty concentrating, being overly alert, and easily startled) could accurately predict patients with moderate/severe disability at 12 months. It is also important to predict patients who will recover well as these patients will likely require less intensive intervention. Initial Neck Disability Index scores of ≤32% and age ≤35 years predicted full recovery at 12 months post-injury. Further evaluation of the clinical prediction rule has shown that it performs comparably to the more generic and commonly used Orebro Musculoskeletal Pain Questionnaire (short form) [69] and may have better specificity (unpublished data). 

The aim of a prognostic clinical prediction rule is to target treatment toward the identified risk groups. For the whiplash clinical prediction rule, it is proposed that patients identified at low-risk of poor recovery require minimal treatment consisting of advice, reassurance and simple exercises [70]. In contrast, it is suggested that patients identified at high risk of poor recovery will require further assessment of potentially contributory factors including psychological distress, nociceptive processing, and neck movement and strength [70]. Whether or not this risk stratified targeted approach results in better patient outcomes is currently being evaluated with no data yet available. 

Clinical message: Risk-stratification may be useful for patients with WAD but further research is required before treatment based on stratification can be recommended.

## 11. Promising Directions for Clinical Practice

Despite the existence of numerous clinical practice guidelines for non-traumatic neck pain and WAD, most recommendations are based on low to moderate quality evidence or on consensus. Clinicians need to be aware of this situation and while broadly following the clinical guideline recommendations, be astute to the individual patient presentation, and adapt their treatments as required. Nonetheless, it is clear that traditional rehabilitation approaches are not very effective and that a step-change is needed to improve this situation. It is difficult to nominate promising directions for clinical practice, as many areas require further research, so the following two sections overlap to some extent. However, some findings if widely adopted in the clinical arena have the potential for an immediate effect to improve health outcomes. Some of these are outlined.

Risk screening or stratification of patients early in development of their neck pain condition followed by provision of a clinical pathway of care based on the patient’s risk of developing chronic/persistent pain. There are several tools available to achieve this—WhipPredict for patients with WAD, the short-form Orebro Musculoskeletal screen for WAD and non-traumatic neck pain, and StartMSK for non-traumatic neck pain (see Table 2 for availability). Research suggests that rehabilitation health care providers are not aware of prognostic indicators and do not use clinical risk screening tools for patients with neck pain [71,72]. The majority of patients will fall into the ‘low risk’ category; in other words, they should recover well with minimal treatment comprising a few sessions of advice, reassurance, and exercise. Those deemed at ‘high risk’ require further evaluation including assessment of psychological factors, nociplastic pain, and more complex movement problems that then could be specifically addressed. Whilst this stratified care model and subsequent care pathway is yet to be fully evaluated for neck pain, there has been substantial research of a similar approach for low back pain. Some trials in low back pain show good effect [73] but others have been more equivocal [74]. In this latter trial conducted in the USA, the intervention resulted in use of the STarT Back risk screening tool, but it did not change health care provider treatment decisions. Some reasons for this proposed by the researchers were unacceptability to clinicians, inadequate leadership and system support, ineffective implementation, and inadequate potency [74]. Neck pain researchers should take note of these issues and they could be addressed in risk-stratified neck pain trials.

The development of more horizontal across discipline (versus vertical siloed uni-discipline) skills for rehabilitation professionals. It is clear that, like all musculoskeletal conditions, neck pain is heterogeneous and the management of particularly the ‘high risk’ patient group requires skills that cut across various disciplines. An example of cross discipline skills is the utilisation of physiotherapists to deliver psychological type treatments. With respect to the targeting of psychosocial risk factors in high risk patients, it has been previously argued in this review that individual specificity will be required, with preliminary evidence showing a broader approach may not be as effective. If further research supports this tenet, then rehabilitation professionals will need to upskill in the identification and management of psychosocial risk factors. At least anecdotally, there seems to be some resistance to this but the evidence is strong that psychosocial factors pay a role in musculoskeletal outcomes and rehabilitation professionals including physiotherapists are well positioned to deliver care that may be considered outside their traditional realm. One cautionary note. It is not advocated that rehabilitation professionals deliver care to patients with a psychopathology such as severe depression or posttraumatic stress disorders and these patients will need referral to a mental health professional. Rehabilitation professionals will require skills in assessment of patient mental health so that appropriate referral can be initiated. The upskilling of the rehabilitation workforce should commence from early undergraduate training and this may be already happening in some locations but is lagging behind in others [78]. 

Provide advice and reassurance that is consistent with identified patient needs. Qualitative research has begun to identify the needs of people with neck pain with respect to the information they are seeking and these findings should lay the foundation for future trials. Some of the factors emerging when talking to people with neck pain may not be traditionally included in advice and reassurance delivered by rehabilitation health care providers. An example is concerns around treatment costs and insurance claims reported by patients with WAD [26]. Psychological distress associated with claims processes have been shown to interfere with recovery and quality of life [79]. Providing information to patients regarding these processes may assist recovery. Another example would be information about likely prognosis nominated as important by patients [76], but which health care providers may not usually provide or be uncomfortable with how to deliver this information to patients. We have consistently found that physiotherapists are not well aware of the consistent predictors of poor recovery after whiplash injury [71,80], so may not routinely assess for such factors with the view of gauging prognosis. There are clinical prediction rules available to identify patients both at risk of poor recovery and those that will recover well [67,68,69] but to date the clinical uptake of these has been slow. This may require more effective knowledge transfer and potentially a cultural change within the physiotherapy profession.

## 12. Promising Directions for Research 

Exercise is the staple approach for most rehabilitation professionals, but further evidence is required to guide this key area of management. The role of exercise in managing neck pain should be clarified including the comparisons of different exercise modalities (strengthening, muscle control, aerobic, and so forth) and dosages. The rationale underpinning the use of exercise is to specifically target underlying physical impairments, which will then impact on pain and disability [81]. However, this assumption has been challenged. In a systematic review for low back pain, little correlation between improvements in clinical outcomes such as pain and disability and improvements in physical function with exercise were found [82]. Similar results were found in a review of neck pain [83] and in a RCT of hip pain [84]. The results of both reviews suggest that improvements in self-reported clinical measures with exercise may be more associated with other factors such as psychological and/or central nervous system responses than by the rectification of specific physical impairments. Certainly, it is clear that exercise exerts central inhibitory effects termed exercise induced hypoalgesia (EIH) [85] and that EIH is impaired in some people with chronic pain including neck pain [35,86]. Further research is required to understand central nervous system responses to exercise in chronic pain and how it may be possible to enhance the EIH response to improve patient outcomes [87]. 

Physical rehabilitation is not the only treatment approach to show mostly small to moderate effects on outcomes. Psychological treatments for chronic musculoskeletal pain show similar effect sizes despite a large number of clinical trials available [88]. Few trials have investigated psychological treatments for neck pain, but based on the chronic pain literature as a whole, it would be expected that similar small effects would also be found. Interestingly, recent calls have been made from the psychology field to consider the body as well as the brain when considering painful conditions. These authors call this ‘embodied pain’ defined by the premise that cognition extends beyond the brain so that an ever-changing body is at the core of how experiences are shaped such as by the unconscious workings of the immune system or the collaborative efforts made to avoid movement [89]. Whilst it is very early days, efforts to combine psychological treatment with physical exercise (“body”) treatment for neck pain may show greater effects than either treatment alone [21], warranting further investigation.

Many reviews of musculoskeletal pain call for further research to identify who does or does not respond to treatment [90,91]. It would be fair to say that little progress has been made in this direction for any condition including neck pain. To date, clinical trials have not been able to identify factors associated with treatment response. An early under-powered RCT of exercise for chronic WAD reported that patients with cold and mechanical hyperalgesia were less likely to respond to exercise [65], but this result could not be replicated in a larger adequately powered study [66]. Similarly, no trials investigating moderating factors on treatment effect for non-traumatic neck pain were identified. Such knowledge would facilitate more individualized patient care. 

The development and testing of innovative methods to include lifestyle interventions for neck pain is needed. It is known that many lifestyle factors are associated with chronic musculoskeletal pain including physical activity [60], sleep [92], smoking [93], stress [94,95], and possibly diet [96]. At the current time, it is not known if addressing these factors will prevent the development of neck pain or the transitions to recovery once an injury has occurred [97].

Other areas requiring research and clinical attention include the development and implementation of modern methods of treatment delivery, in order to enhance access to treatment. Technology assisted methods, such as telehealth, have been successfully used in the rehabilitation of conditions, such as stroke [98] and post orthopaedic surgery [99], and show promise for the delivery of psychological treatment. Such approaches have not been readily taken up in musculoskeletal pain practice, perhaps due to the perception that passive hands-on treatment is required. However, the evidence dictates that passive treatment is not essential for neck pain conditions. Rather, active treatment approaches that improve patient self-efficacy are preferable due the stronger evidence base and these could be delivered in more innovative ways than the traditional face-to-face sessions.

Key messages:

- Strengthening exercises of the neck and upper quadrant have a moderate effect on neck pain in the short-term. This conclusion is based on moderate quality evidence.
- Other exercise approaches demonstrate small effects based mostly on low quality evidence.
- Reassurance/advice/education generally show small effects based on low to moderate quality evidence.
- Psychological treatments alone have small effects based on very low to moderate quality evidence.
- Combined psychological and physical treatments delivered by physiotherapists may be more effective.
- Clinical guidelines are mostly based upon low to moderate quality evidence or consensus, so future research will likely change these conclusions.
- Clinicians should consider the limitations of the evidence regarding rehabilitation for chronic neck pain, and as such broadly follow clinical guidelines; however, adapt treatment to each patient as appropriate.

Finally, and by no means the least important, is that improved understanding of biological processes underlying neck pain is required as this will provide direction for new and innovative treatments. There is evidence available indicating impaired immune responses in WAD and neck arm pain [100], some evidence of quantitative imaging biomarkers [101] and emerging data of genetic variants of stress biomarkers associated with non-recovery after whiplash injury [102].

## Figures and Tables

**Table 1:** Best evidence summary—systematic reviews and RCTs if conducted after publication of a relevant systematic review.

Intervention	Target Population	Level of Evidence	Quality of Evidence	Effect Size	Site of Care	Rehabilitation Professions	Key References and/or Treatment Manuals
Reassurance, advice, education							
Video in ED focussing on activation	WAD (*n* = 348)	Level I [9]	Moderate	Small effect compared to no treatment at intermediate follow-up, RR 0.79 (0.59 to 1.06), NNT:23	ED	All	See systematic review
WAD information pamphlet	WAD (*n* = 102)	Level I [9]	Low	No effect compared to generic advice	ED	All	See systematic review
Booklet or email	NTNP (*n* = 64)	Level 1 [10]	Moderate	No effect compared to massage or exercise	Primary	All	See systematic review
Booklet/neck school	NTNP (*n* = 411)	Level 1 [11]	Very low to low	No effect	Primary and secondary	All	
Exercise							
Strengthening (upper quarter)	WAD and NTNP (*n* = 241)	Level I [12]	Moderate	Moderate to large at short-term follow-up, SMD (pain) −0.71 (−1.33 to −0.10)	Primary and secondary	Exercise professionals	See systematic review
	Office workers with neck pain (*n* = 605)	Level I [13]	Moderate	Moderate effect vs. no intervention, SMD pain = 0.59 (0.29 to 0.89)	Workplace		
Endurance training (upper quarter)	WAD and NTNP (*n* = 198)	Level I [12]	Moderate	Small at short-term follow-up	Primary and secondary	Physiotherapists	See systematic review
Muscle control (stabilisation)	WAD and NTNP (*n* = 71)	Level I [12]	Moderate	Small at intermediate-term follow-up Small to moderate effect on pain in the short to intermediate term (SMD pain −0.59 (95% CI: −0.97 to −0.20))	Primary and secondary	Physiotherapists	See systematic review
	NTNP (*n* = 174)	Level 1 [14]	Low to moderate	Small effect on disability (SMD disability −0.44 (95% CI: −0.81 to −0.08)) vs. other treatments	Primary and secondary	Physiotherapists	
Stretching (neck & shoulder)	Workers (*n* = 96)	Level II [15]	Pedro (8/10)	Small effect on pain & disability compared to ergonomic advice (−1.4; 95% CI −2.2 to −0.7 for pain; −4.8; 95% CI −9.3 to −0.4 for disability)	Work place	Exercise professionals	Exercise protocol available at [15]
Eye-neck co-ordination/proprioception	WAD & NTNP (*n* = 103)	Level I [16]	Very low	Small effect on pain MD: −1.6 (−3.6 to 0.3) compared to no exercise Meta-analysis for other outcomes could not be conducted	Primary and Secondary	Physiotherapists	See systematic review
Qigong	WAD and NTNP (*n* = 191)	Level I [12]	Moderate	Small at intermediate-term follow-up	Primary and secondary	Exercise professionals	See systematic review
Yoga	NTNP (*n* = 686)	Level I * (high heterogeneity) [17]	Moderate	Moderate effect on pain and disability vs. various other treatments including exercise, SMD pain = −1.13 (−1.60 to −0.66), SMD disability −0.92 (−1.38 to 0.47)	Primary and secondary	Exercise professionals	See systematic review
General exercise	WAD, NTNP, workers (*n* = 386)	Level I [13,18]		No effect	Primary and secondary	Exercise professionals	See systematic review
Psychological treatments alone (CBT)	WAD and NTNP (*n* = 168)	Level I [19]	Very low to moderate	Small effect on pain and disability when compared to no treatment, SMD pain = −0.58 (−1.01 to −0.16), SMD disability = −0.61 (−1.21 to −0.01)	Primary and secondary	Psychology professionals	See systematic review
Combined psychological and physical treatments delivered by physiotherapists	WAD (*n* = 211)	Level I * (high heterogeneity) [20]	Moderate quality	No effect on pain and disability	Primary	Physiotherapists	See systematic review
				Medium effect on fear of avoidance			
	Acute WAD (*n* = 108)	Level II (RCT) [21]	NA Pedro (8/10)	Medium to large effect on pain related disability compared to exercise only		Physiotherapists	Treatment protocols available at [21]
Exercise and manual therapy	WAD and NTNP (*n* = 345)	Level I [22]		No effect compared to exercise alone	Primary and secondary		

Levels of Evidence were defined as per the Oxford Centre for Evidence Based Medicine [23]. Quality of Evidence as per reported in Systematic reviews or per Pedro scale for RCTs. * Indicates systematic reviews with high heterogeneity indicating caution is required with interpretation of results. WAD: whiplash associated disorders; NTNP: non-traumatic neck pain; ED: emergency department; SMD: standardised mean difference; MD: mean difference; NNT: number needed to treat.

**Table 2:** Promising directions for clinical practice.

Treatment Approach	Resources
Risk screening/stratification of patients to determine risk of poor or delayed recovery	WhipPredict (Whiplash Clinical Prediction Tool) [67,68]. Electronic version available at https://recover.centre.uq.edu.au/research/clinician-resources Hard copy available on author request Orebro Musculoskeletal Screening Tool Short-Form [69] Available at https://www.cesphn.org.au/documents/filtered-document-list/204-oerebro-musculoskeletal-pain-screening-questionnaire/file StartMSK [75]. Available at https://www.keele.ac.uk/startmsk/moreaboutthetool/
Clinical pathways of care based on risk stratification	Under evaluation. Protocol available at [70]
Development of skills of rehabilitation professionals to integrate some psychological treatments into standard physical rehabilitation	There are various protocols available but few that are specific to neck pain The integration of a psychological treatment targeting stress related symptoms in people with acute WAD is available at the following reference [21]
Provide advice and reassurance to patients that is more targeted to their needs	Preliminary findings of the needs of patients with WAD and neck pain [26,76,77]
